# Leaf apoplastic proteome composition in UV-B treated *Arabidopsis thaliana* mutants impaired in extracellular glutathione degradation

**DOI:** 10.1016/j.dib.2015.12.005

**Published:** 2015-12-17

**Authors:** A. Masi, A.R. Trentin, G. Arrigoni

**Affiliations:** a DAFNAE, University of Padova, Italy; b Proteomic Center, University of Padova, Italy; c Department of Biomedical Sciences, University of Padova, Italy

**Keywords:** Apoplast, Environmental stress, Gamma-glutamyl transferase, Glutathione, Oxidative stress, ROS

## Abstract

In plants, environmental perturbations often result in oxidative reactions in the apoplastic space, which are counteracted for by enzymatic and non-enzymatic antioxidative systems, including ascorbate and glutathione. However, the occurrence of the latter and its exact role in the extracellular space are not well documented. In *Arabidopsis thaliana*, the gamma-glutamyl transferase isoform GGT1 bound to the cell wall takes part in the so-called gamma-glutamyl cycle for extracellular glutathione degradation and recovery, and may be implicated in redox sensing and balance.

In this work, oxidative conditions were imposed with UV-B radiation and studied in redox altered *ggt1* mutants. Elevated UV-B has detrimental effects on plant metabolism, plasma membranes representing a major target for ROS generated by this harmful radiation. The response of *ggt1* knockout *Arabidopsis* leaves to UV-B radiation was assessed by investigating changes in apoplastic protein composition.

We then compared the expression changes resulting from the mutation and from the UV-B treatment. Rearrangements occurring in apoplastic protein composition suggest the involvement of hydrogen peroxide, which may ultimately act as a signal. Other important changes related to hormonal effects, cell wall remodeling, and redox activities are also reported. We argue that oxidative stress conditions imposed by UV-B and by disruption of the gamma-glutamyl cycle result in similar stress-induced responses, to some degree at least. Data shown here are associated with the article from Trentin et al. (2015) [1]; protein data have been deposited to the PRIDE database (Vizcaíno et al., 2014) [2] with identifier PXD001807.

**Specifications Table**

**Table:** 

Subject area	Plant Physiology and Biochemistry
More specific subject area	Glutathione metabolism
Type of data	MS data and annotations, spectrophotometric and chromatographic data
How data was acquired	i-TRAQ labelled peptides were analysed using mass spectrometry (LTQ Orbitrap, Thermo Scientific)
Data format	Analysed output data
Experimental factors	Apoplastic fluids (or ECWF, Extra-Cellular Washing Fluid) were obtained by the infiltration/centrifugation method
Experimental features	Depending on the purpose of analysis, different infiltration buffers were used for antioxidant measurements or proteome composition analysis.
Data source location	NOT APPLICABLE
Data accessibility	Proteomic data are stored and available in a public repository (PRIDE database, PXD001807, url: 〈http://proteomecentral.proteomexchange.org/dataset/PXD001807〉)

**Value of the data**

• Apoplastic proteomes from *A. thaliana* wt and ggt1- knockout mutants are compared for functional characterization of the cell-wall bound gamma-glutamyl transferase/transpeptidase GGT1 enzyme.
• Effects of UV-B radiation on the extracellular protein composition are also reported.
• Quantitative proteomics was performed by iTRAQ labelling.
• Results point to a role for apoplastic GGT1 in redox sensing/signaling.

## 1 Experimental design

A major aim of this analysis was to obtain information on the significance of the enzyme gamma-glutamyl transferase (GGT) in the response to oxidative conditions. Since the apoplastic isoform GGT1 is extracellular and cell-wall bound, we hypothesised that disrupting this enzyme׳s activity would result in altered redox conditions in the apoplast, that may affect the overall response to oxidative stress conditions starting from the apoplast. To this regard, UV-B radiation is known to induce oxidative damage to plasma membranes and originate ROS in the apoplast.

Therefore, we used a *ggt1* mutant line that had been previously characterized [3,4], and imposed a UV-B treatment. In this way, we generated four experimental conditions: 1) untreated, wildtype; 2) untreated, *ggt1* mutant; 3) UV-B treated, wildtype; and 4) UV-B treated, *ggt1* mutant.

Finally, we obtained the extracellular washing fluid (ECWF) with the aim to gain the following information: i) the effect of UV-B treatment on each genotype; ii) differential apoplastic protein composition in *ggt1 vs* . wildtype; iii) possible differences in the behavior of the *ggt1* mutant and the wildtype under UV-B.

## 2 Materials and methods

### 2.1 Plant materials and growth conditions

Seeds of *Arabidopsis thaliana* wildtype and a *ggt1* knockout mutant line, both Columbia ecotype (Col-0), were obtained from the Nottingham A. thaliana Stock Centre (〈http://nasc.nott.ac.uk〉; polymorphism SALK_080363) [5]. The UV-B treatment was applied for 8 h at the beginning of the light period, to plants at the stage of fully expanded rosette. The growth chamber settings were: 12/12 h light/dark cycle, 21/21 °C temperature, 300 µmol m^−2^ s^−1^ photosynthetically active radiation, and 60% relative humidity. The radiation was provided by two Philips TL40W/12 lamps with an intensity of 8.3 kJ m^−2^ d^−1^ (UVBBE, biologically effective UV-B), measured on the level of the plants.

### 2.2 Apoplastic fluid extraction

**Fig. 1:**
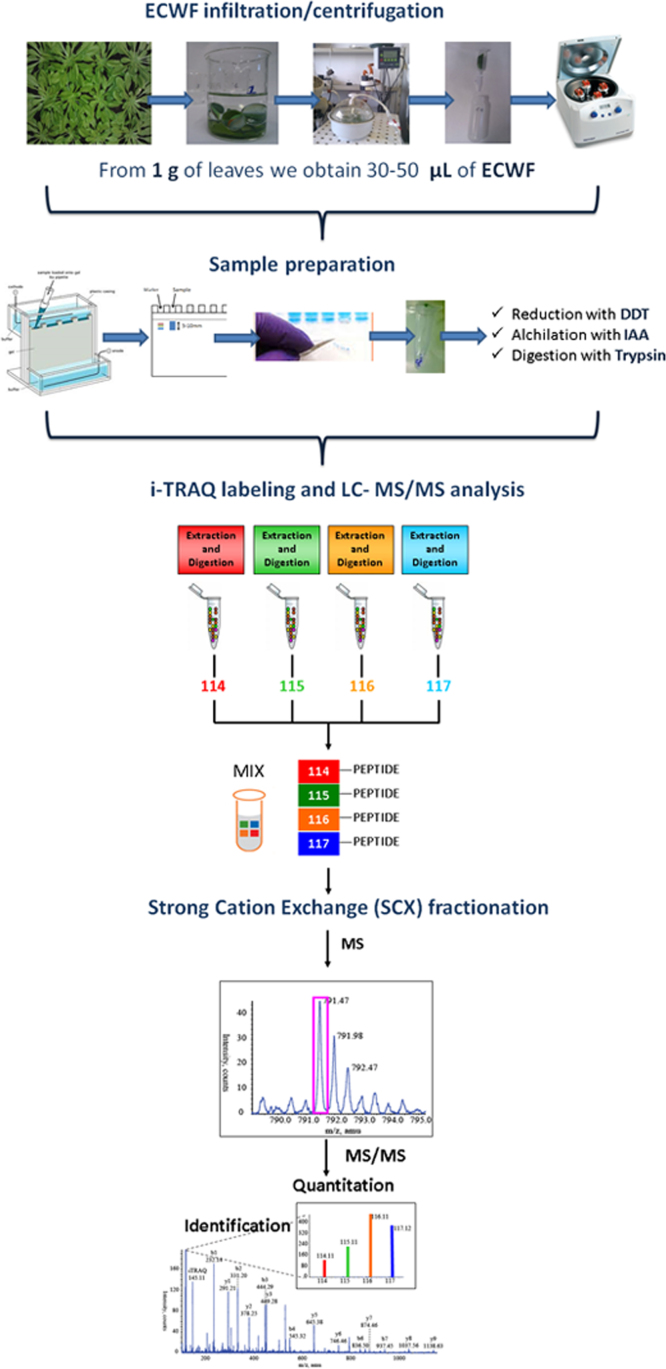
Experimental workflow. Following apoplastic fluid extraction by the infiltration/centrifugation protocol (see Section 2 for details), electrophoresed proteins were reduced, alkylated and digested with trypsin. Peptides from the four experimental conditions were then labeled with iTRAQ, pooled and analysed by LC–MS–MS for simultaneous quantitation and identification.

Extracellular washing fluids (ECWF) were extracted by vacuum infiltration (Fig. 1). About 1 g of mature fresh leaves were cut from 4 to 5 Arabidopsis rosettes, rinsed, immersed in infiltration buffer and vacuum-infiltrated for 10 min at 20 kPa.

The composition of infiltration buffer was: KH_2_PO_4_ 50 mM, KCl 0.2 M and PMSF 1 mM, pH 6.2. After infiltration, the leaves were blot-dried, weighed and placed vertically in a 5 ml syringe. The syringes were placed in tubes and centrifuged at 200*g*, 4 °C for 20 min. Apoplastic fluids were collected in eppendorf tubes placed in the bottom of the large tubes. Typically, 30–50 µL of ECWF was retrieved at the end of this procedure.

### 2.3 Proteome analysis

#### 2.3.1 Protein sample preparation and in situ digestion

Proteins obtained from ECWF were quantified by bicinchoninic acid spectrophotometric assay; 50 µg of proteins were loaded into a homemade 11% SDS gel and the electrophoretic run was stopped as soon as the protein extracts entered the running gel. The significance of this preliminary step is to remove salts and any other possible interfering compounds from the sample. Bands were then excised and washed several times with 50 mM TEAB (triethylammonium bicarbonate) and dried under vacuum after a short acetonitrile wash. Cysteines were reduced with 10 mM dithiothreitol (in 50 mM TEAB) for 1 h at 56 °C, and alkylated with 55 mM iodoacetamide (in 50 mM TEAB) for 45 min at room temperature in the dark. Gel pieces were then washed with alternate steps of TEAB and acetonitrile, and dried under vacuum. Proteins were *in situ* digested with sequencing grade modified trypsin (Promega, Madison, WI, USA) at 37 °C overnight (12.5 ng/μL trypsin in 50 mM TEAB). Peptides were extracted with three steps of 50% acetonitrile in water. 1 µg of each sample was withdrawn to check digestion efficiency using LC–MS/MS analysis, and the remaining peptide solution was dried under vacuum.

#### 2.3.2 iTRAQ labeling and peptide fractionation

Peptides were labeled with iTRAQ reagents (ABSciex) according to the manufacturer׳s instructions. They were labeled with the four iTRAQ tags using a Latin panel strategy: wt UV-B, ggt1 UV-B, wt ctrl and ggt1 ctrl were labeled respectively with 114, 115, 116 and 117 tags in the first replicate; 115, 116, 117, 114 tags in the second and 116, 117, 114, 115 tags in the third replicate. Prior to mixing the samples in a 1:1:1:1 ratio, 1 μg of each sample was analyzed separately to check label efficiency by LC–MS/MS analysis. In these cases, iTRAQ labeling was set as a variable modification in the database search, while the other settings were as reported below (Section 2.3.5). This step of quality control is particularly useful to highlight possible partial/incomplete labeling that might affect the final quantification outcome. If a relevant number of peptides are identified as being not correctly modified, the labeling step can be potentially repeated. Our control of labeling efficiency showed that all the peptides were correctly identified as being iTRAQ-modified at the N-terminus and at each lysine residue. Only at this point the samples were pooled and dried under vacuum.

#### 2.3.3 Strong cation exchange fractionation

To reduce complexity and increase the number of protein and peptide identifications, the samples were subjected to a step of peptide fractionation by strong cation exchange (SCX) chromatography on a SCX cartridge (AB Sciex, MA, USA). The labeled samples were dissolved in 500 µL of buffer A (10 mM KH_2_PO_4_, 25% acetonitrile, pH 3.0) and loaded onto the cartridge using a syringe pump at a flow rate of 50 µL/min. After 3 washes with 500 µL of buffer A, peptides were eluted in a stepwise manner with 500 µL of the following concentrations of KCl in buffer A: 25, 50, 100, 200, and 350 mM. The volume of each fraction was reduced under vacuum to remove acetonitrile. Samples were desalted using C18 cartridges (Sep-Pack, C18, Waters, Milford, MA, USA) according to the manufacturer׳s instructions and dried under vacuum.

#### 2.3.4 LC–MS/MS analysis

Samples were suspended in 0.1% formic acid/3% acetonitrile and analyzed by LC–MS/MS. The MS analyses were conducted with a LTQ-Orbitrap XL mass spectrometer (Thermo Fisher Scientific, Pittsburgh, CA, USA) coupled online with a nano-HPLC Ultimate 3000 (Dionex-Thermo Fisher Scientific). Samples were loaded onto a trap-column (300 μm id, 300 A, C18, 3 μm; SGE Analytical Science) at a flow rate of 8 μL/min, washed for 6 min and then transferred to a homemade 10 cm chromatographic column packed in-house into a pico-frit (75 µm id, 10 mm tip, New Objectives) with C18 material (ReproSil, 300 Å, 3 μm).

**Fig. 2:**
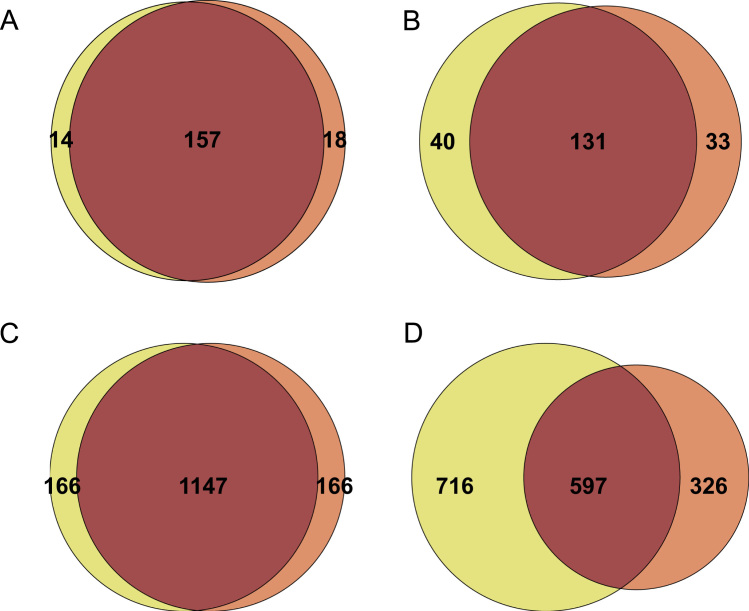
Effect of the application of the static exclusion list. A strong overlap of data at the protein and peptide level is observed when the same sample is analyzed twice under identical conditions (Panels A and C respectively). When a static exclusion list is generated and included in the instrumental method, the overlap of data is significantly reduced at the protein level (Panel B) and overall at the peptide level (Panel D).

Peptides were eluted with a linear gradient of acetonitrile/0.1% formic acid from 3% to 50% in 90 min at a flow rate of 250 nL/min. Spray voltage was set at 1.3–1.4 kV, capillary temperature at 200 °C, capillary voltage at 49 V, and tube lens at 120 V. According to the method described by Köcher et al. [6], the instrument performed a full scan at high resolution (60,000) on the Orbitrap, with a mass range of 300–1600 Da, followed by MS/MS scans on the three most intense ions with CID fragmentation on the linear trap. Only for quantification purposes MS/MS scans were performed on the same ions with higher energy collision dissociation (HCD) fragmentation on the Orbitrap (with a resolution of 7500). HCD fragmentation allows to obtain low mass range data suitable for protein quantification. In order to favor the release of reporter ions from the iTRAQ tags and obtain a more reliable quantification, a normalized collision energy of 50 was set for HCD fragmentation. Maximum injection time was set to 100 ms for MS/MS spectra acquired in the linear ion trap, while for full MS and HCD MS/MS spectra was set to 500 ms and 1000 ms respectively. AGC was 5×10^5^ for full MS spectra and 1×10^4^ and 2×10^5^ for CID and HCD spectra respectively. For both CID and HCD fragmentation repeat count was set to 1, while repeat duration and exclusion duration were set to 30 s and 180 s respectively. All ions with charge state +1 or unassigned were excluded by the process of precursor selection. The minimum threshold for triggering the MS/MS acquisition was set to 500 counts. Isolation width was 2 m/z, both for CID and HCD fragmentation methods. For CID, normalized collision energy was set to 35, with activation Q of 0.250 and activation time of 30 ms. As mentioned above, for HCD fragmentation the normalized collision energy was set to 50 to maximize the intensity of the reporter ions. The peptides reliably identified in each sample by the database search (as specified below) were inserted in a static exclusion list that was used to perform (under the same chromatographic and instrumental conditions) a second LC–MS/MS run for each sample fraction. Analyzing the same sample twice with the application of the excluding list allows to increase the number of peptide identifications, as well as the number of protein IDs and sequence coverage. As shown in Fig. 2, when the same sample is analyzed twice under identical conditions, the very large majority of proteins and peptides are in common between the two analyses (panels A and C, respectively). When the static excluding list is applied during the second analysis, both protein and peptide identifications increase (panels B and D) and, as expected, the effect is much more evident at the peptide level, while for the proteins the improvement is more evident at the level of sequence coverage. By looking more in detail at the results obtained with the application of the excluding list, we could observe that for about 30% of the peptides that are identified as being in common, the MS/MS spectra were acquired from the same peptides in different charge states. Obviously, the application of the static excluding list does not result in a complete lack of overlapping data, but these results clearly show that it is an efficacious method to reduce the undersampling effect in complex samples.

#### 2.3.5 Database search and protein quantification

The raw LC–MS/MS files were analyzed using the software Proteome Discoverer 1.4 (Thermo Fisher Scientific), connected to a Mascot Search Engine server (version 2.2.4, Matrix Science, London, UK). The spectra were searched against a ARATH UniProt protein database (version 2014.04.16, 33,353 sequences, 13,619,890 residues, www.uniprot.org [7]) using a MudPit protocol: all raw files acquired for each biological replicate were processed together, being fractions of the same original sample. Enzyme specificity was set to trypsin with two missed cleavages, and peptide and fragment tolerance was set to 10 ppm and 0.6 Da, respectively. Methylthiocysteine, 4-plex iTRAQ at the N-terminus and Lys were set as fixed modifications, except for the quality control step, where iTRAQ labeling was set as variable modification, as specified above (see Section 2.3.2). In all cases, Methionine oxidation was selected as variable modification. Percolator in combination with the search against a randomized database was used to assess false discovery rates (FDR). Data were pre-filtered to exclude MS/MS spectra containing less than 5 peaks or with a total ion count below 50. The protein relevance threshold was set to 20 and the peptide cutoff score was set to 10. Only proteins quantified with at least 2 unique peptides of rank 1 and with a 99% confidence (*q* value <0.01) were considered as positive identifications. Only unique peptides were used for quantification. Quantification data were corrected by normalizing the results on the median value of all measured iTRAQ reporter ratios.

The data deposited in PRIDE database (PXD001807) [2] consist in all raw files acquired for each biological replicate and divided according to the SCX fractionation that was performed for each replicate. The total list of proteins and peptides identified in the study is reported as supplementary material in [1]. The mean value of at least 2 biological replicates was used to express the final quantifications that are reported according to the following ratios: wt (UV-B/ctrl), ggt1 (UV-B/ctrl), ctrl (ggt1/wt) and UV-B (ggt1/wt). A two-tailed *Z* test was performed and only proteins that were quantified with a confidence value of *p*<0.05 were retained in the final list. The variations were further restricted to proteins exhibiting an expression fold change of at least ±50% (1.5 for upregulated and 0.68 for downregulated proteins).

## 3 Data

A summary of the main information regarding number of search inputs, PSMs, peptide IDs, and protein IDs is reported in Table 1. As it is possible to observe, for one of the samples only two SCX fractions were obtained. For this sample the amount of apoplastic proteins that were retrieved from the procedure described above (Section 2.2) was too low to allow a deeper fractionation. The number of protein and peptide IDs from this biological replicate reflect the fact that a lower amount of material was analyzed.

For each of the biological replicates the number of SCX fractions performed, the number of search inputs, PSMs, peptide and protein IDs are reported.

Nevertheless, LC–MS/MS analyses led to the identification of a total of 329 proteins; of them, 208 were found in at least two biological replicates. We restricted our analysis to the 118 proteins that were either apoplastic or unlocalized (based on the Gene Ontology assignment for cellular compartmentalization; 〈www.uniprot.org〉 [8], accounting for approximately 57% of the total. The choice of including unlocalised proteins may represent a potential risk of considering as apoplastic some proteins that are not; however, we decided to be less conservative since several evidences in literature point to the occurrence of unconventional secreted proteins that are not predicted as such by bioinformatics tools [9], [10]. The variations considered were further restricted to proteins exhibiting an at least ±50% fold change in expression.

**Fig. 3:**
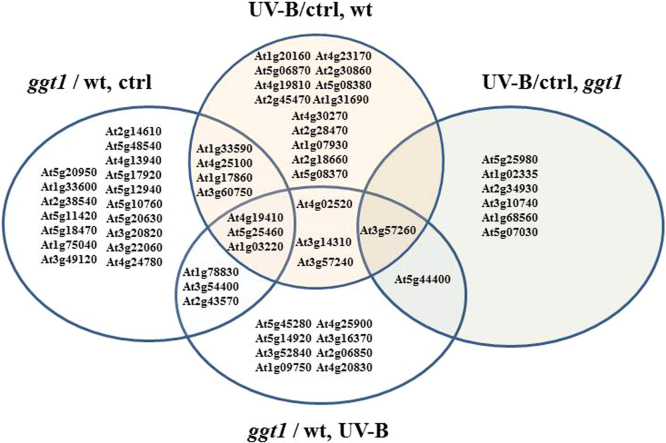
Venn diagram showing the apoplastic proteins that are altered (±50% fold change) in the *ggt1* mutant compared to the wildtype (*ggt1*/wt, ctrl), following UV-B treatment in the wildtype (UV-B/ctrl, wt) or in the *ggt1* mutant (UV-B/ctrl, *ggt1*), or in UV-B treated *ggt1 vs* . wildtype mutant plants (*ggt1*/wt, UV-B).

Differentially expressed proteins are listed in Table 2, Table 3, Table 4, Table 5 and compared in the Venn diagram shown in Fig. 3. This diagram shows that a subset of proteins are altered both as a consequence of the *ggt1* mutation, and of the UV-B treatment. These proteins are involved in ROS metabolism (as superoxide dismutase At4g25100) and in cell wall remodeling; one is a Leucine-rich repeat-containing protein (At1g33590), which is associated to the plasma membrane and is likely to act as a receptor.

This comparative analysis lead to the hypothesis that the gamma-glutamyl cycle may participate in ROS-mediated environmental stress sensing, by transferring redox signals arising in the apoplast to the inner compartments [1], [4], [11].

## Figures and Tables

**Table 1:** Summary of data obtained from LC–MS/MS and database search.

	Replicate 1	Replicate 2	Replicate 3
Number of fractions (.raw files)	10	10	2
Search inputs	32,355	37,681	9659
PSMs	3746	2602	463
Peptides	2020	1572	341
Proteins	435	323	84
Protein groups	310	241	64
Unique peptides	3145	2256	422
Not unique peptides	262	221	23
Not quantified peptides	50	11	5
Redundant peptides	289	114	13
CID identifications	3440	2486	448
HCD identifications	306	116	15

**Table 2:** Expression change values in apoplastic proteins in ggt1 *vs*. wildtype plants under physiological conditions.

**UniProt ID/AC**	**Locus Name**	**ggt1/Col O**	**Description**	**% Cov.**	**# Pep**
P28493	At1g75040	0.30	Pathogenesis-related protein 5	60.7	11
O24603	At2g43570	0.34	Chitinase class 4-like protein	28.5	7
P33154	At2g14610	0.34	Pathogenesis-related protein 1	18.0	3
Q42589	At2g38540	0.42	Non-specific lipid-transfer protein 1	43.2	6
Q9LEW3	At5g10760	0.44	Aspartyl protease family protein	3.5	2
Q9LMU2	At1g17860	0.48	uncharacterized protein	57.1	11
F4HR88	At1g33590	0.48	Leucine-rich repeat-containing protein	59.3	23
Q9LRJ9	At3g22060	0.49	Cysteine-rich repeat secretory protein 38	36.1	10
Q9LV60	At5g48540	0.50	Cysteine-rich repeat secretory protein 55	39.2	10
Q9LXU5	At5g12940	0.51	Leucine-rich repeat-containing protein	34.5	12
P94072	At5g20630	0.52	Germin-like protein subfamily 3 member 3	29.4	4
Q94K76	At5g18470	0.53	Curculin-like (Mannose-binding) lectin family protein	10.4	4
Q9LYE7	At5g11420	0.55	uncharacterized protein	34.2	15
Q9SMU8	At3g49120	0.56	Peroxidase 34	23.5	8
Q9ZVA2	At1g78830	0.57	Curculin-like (Mannose-binding) lectin-like protein	42.4	19
Q94F20	At5g25460	0.58	uncharacterized protein	42.3	17
Q9FW48	At1g33600	0.58	Leucine-rich repeat-containing protein	43.3	19
Q9M2U7	At3g54400	0.64	Aspartyl protease family protein	23.1	11
Q8W112	At5g20950	0.65	Beta-D-glucan exohydrolase-like protein	16.5	11
Q9ZVS4	At1g03220	0.66	Aspartyl protease-like protein	27.7	11
Q9LT39	At3g20820	0.67	Leucine-rich repeat-containing protein	49.9	15
Q9C5M8	At4g24780	0.68	Probable pectate lyase 18	12.3	3
Q940J8	At4g19410	0.68	Pectinacetylesterase family protein	63.2	21
O23255	At4g13940	1.5	Adenosylhomocysteinase 1	24.74	12
F4JRV2	At4g25100	1.7	Superoxide dismutase	12.4	3
F4JBY2	At3g60750	2.2	Transketolase	29.1	17
O50008	At5g17920	2.4	5-methyltetrahydropteroyltriglutamate--homocysteine methyltransferase	36.21	28

**Table 3:** Expression change values in apoplastic proteins in UV-B treated *vs*. untreated wildtype plants.

**UniProt ID/AC**	**Locus Name**	**ggt1/Col O**	**Description**	**% Cov.**	**# Pep**
F4HR88	At1g33590	0.55	Leucine-rich repeat-containing protein	59.3	23
O81862	At4g19810	0.55	Class V chitinase	18.5	5
Q9LMU2	At1g17860	0.57	uncharacterized protein	57.1	11
F4IAX0	At1g31690	0.57	Putative copper amine oxidase	7.8	4
Q9M5J8	At5g06870	0.57	Polygalacturonase inhibitor 2	20.0	6
B9DGL8	At5g08370	0.58	Alpha-D-galactoside galactohydrolase 2	25.7	11
F4HSQ4	At1g20160	0.61	Subtilisin-like serine endopeptidase-like protein	5.5	3
F4IIQ3	At2g28470	0.62	Beta-galactosidase	11.2	10
Q9ZVS4	At1g03220	0.65	Aspartyl protease-like protein	27.7	11
Q94F20	At5g25460	0.66	uncharacterized protein	42.3	17
Q9FT97	At5g08380	0.68	Alpha-galactosidase 1	34.9	13
Q940J8	At4g19410	0.68	Pectinacetylesterase family protein	63.2	21
O65469	At4g23170	1.5	Putative cysteine-rich receptor-like protein kinase 9	14.7	4
O49006	At3g14310	1.5	Pectinesterase/pectinesterase inhibitor 3	6.9	4
P24806	At4g30270	1.6	Xyloglucan endotransglucosylase/hydrolase protein 24	24.5	7
F4J270	At3g57240	1.7	Beta-1,3-glucanase 3	51.3	13
Q9ZV52	At2g18660	1.8	EG45-like domain containing protein 2	23.9	3
P46422	At4g02520	1.8	Glutathione S-transferase F2	59.4	13
F4JRV2	At4g25100	1.9	Superoxide dismutase	12.4	3
O22126	At2g45470	1.9	Fasciclin-like arabinogalactan protein 8	9.3	4
P33157	At3g57260	2.1	Glucan endo-1,3-beta-glucosidase, acidic isoform	38.4	11
F4JBY2	At3g60750	2.7	Transketolase	29.1	17
O80852-2	At2g30860	2.9	Isoform 2 of Glutathione S-transferase F9	24.7	4
F4HUA0	At1g07930	4.4	Elongation factor 1-alpha	19.9	8

**Table 4:** Expression change values in apoplastic proteins in UV-B treated *vs*. untreated ggt1 mutant plants.

**UniProt ID/AC**	**Locus Name**	**ggt1/Col O**	**Description**	**% Cov.**	**# Pep**
O64757	At2g34930	0.31	Disease resistance-like protein/LRR domain-containing protein	14.6	13
Q9SG80	At3g10740	0.35	Alpha-L-arabinofuranosidase 1	23.6	16
Q9FZ27	At1g02335	0.37	Germin-like protein subfamily 2 member 2	20.6	4
Q9FKU8	At5g44400	0.49	Berberine bridge enzyme	11.0	6
F4K5B9	At5g07030	0.54	Aspartyl protease family protein	30.8	12
Q9S7Y7	At1g68560	0.55	Alpha-xylosidase 1	13.0	10
Q9C5C2	At5g25980	0.61	Myrosinase 2	30.0	14
P33157	At3g57260	0.63	Glucan endo-1,3-beta-glucosidase, acidic isoform	38.4	11

**Table 5:** Expression change values in apoplastic proteins in in ggt1 *vs*. wildtype plants, treated with UV-B radiation.

**UniProt ID/AC**	**Locus Name**	**ggt1/Col O**	**Description**	**% Cov**	**#Pep**
O24603	At2g43570	0.17	Chitinase class 4-like protein	28.5	7
P33157	At3g57260	0.26	Glucan endo-1,3-beta-glucosidase, acidic isoform	38.4	11
Q9SVG4-2	At4g20830	0.43	Isoform 2 of Reticuline oxidase-like protein	40.6	22
F4J270	At3g57240	0.47	Beta-1,3-glucanase 3	51.3	13
P46422	At4g02520	0.51	Glutathione S-transferase F2	59.4	13
O49006	At3g14310	0.55	Pectinesterase/pectinesterase inhibitor 3	6.9	4
Q9LFA6	At3g52840	0.59	Beta-galactosidase 2	10	9
Q940G5	At4g25900	0.61	Aldose 1-epimerase family protein	56.0	14
Q9FKU8	At5g44400	0.68	Berberine bridge enzyme	11.0	6
Q9LU14	At3g16370	1.57	GDSL esterase/lipase APG	34	10
Q94F20	At5g25460	1.59	uncharacterized protein	42.3	17
Q9LFR3	At5g14920	1.80	Gibberellin-regulated protein 14	14.2	5
Q39099	At2g06850	1.83	Xyloglucan endotransglucosylase/hydrolase protein 4	49.3	17
Q940J8	At4g19410	1.88	Pectinacetylesterase family protein	63.2	21
O04496	At1g09750	1.92	Aspartyl protease-like protein	14.0	7
Q9FH82	At5g45280	2.04	Pectin acetylesterase 11	34.5	12
Q9M2U7	At3g54400	2.04	Aspartyl protease family protein	23.1	11
Q9ZVA2	At1g78830	2.32	Curculin-like (Mannose-binding) lectin-like protein	42.4	19
Q9ZVS4	At1g03220	2.50	Aspartyl protease-like protein	27.7	11
